# Working together to increase Australian children’s liking of vegetables: a position statement by the Vegetable Intake Strategic Alliance (VISA)

**DOI:** 10.1017/S1368980023001398

**Published:** 2023-11

**Authors:** David Nicholas Cox, Karen J Campbell, Lynne Cobiac, Claire Gardner, Lucinda Hancock, Gilly A Hendrie, Amber Kelaart, Michelle Lausen, Astrid AAM Poelman, Ros Sambell, Kim M Tikellis, Bonnie Wiggins

**Affiliations:** 1 CSIRO Health & Biosecurity, Adelaide, SA 5000, Australia; 2 Institute for Physical Activity and Nutrition, Deakin University, Melbourne, VIC 3000, Australia; 3 Caring Futures Institute, Flinders University, Bedford Park, SA 5042, Australia; 4 Nutrition Australia (Vic, SA Tas, WA), Melbourne, VIC 3000, Australia; 5 CSIRO Health & Biosecurity, Westmead, NSW 2145, Australia; 6 Edith Cowan University, Joondalup, WA 6027, Australia; 7 Coles Group, Hawthorn East, VIC 3123, Australia

**Keywords:** Vegetables, Child, Learning, Taste perception, Behaviour, Community participation, Pleasure

## Abstract

Children need to be repeatedly and consistently exposed to a variety of vegetables from an early age to achieve an increase in vegetable intake. A focus on enjoyment and learning to like eating vegetables at an early age is critical to forming favourable lifelong eating habits. Coordinated work is needed to ensure vegetables are available and promoted in a range of settings, using evidence-based initiatives, to create an environment that will support children’s acceptance of vegetables. This will help to facilitate increased intake and ultimately realise the associated health benefits. The challenges and evidence base for a new approach are described.

## Graphical abstract

**Figure:**
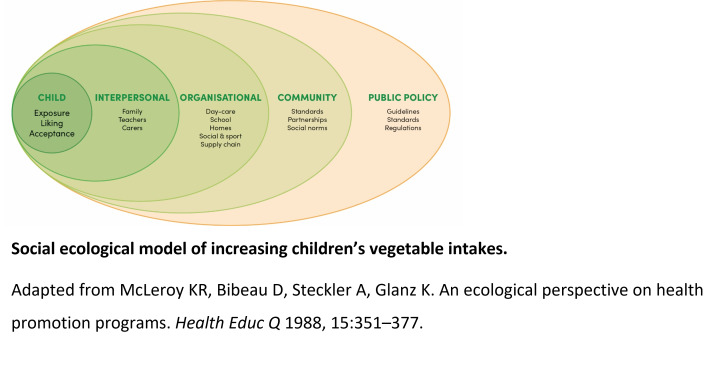

Children’s vegetable intakes tend to be low worldwide and in Australia have stagnated far below recommendations^(1)^. Past efforts to increase intakes have been limited and not adopted at scale^(2,3)^. In this position statement, an alliance of stakeholders, the Vegetable Intake Strategic Alliance (VISA) provides a unified voice in the promotion of evidence-based best practice using a paradigm shift to improve vegetable intakes.

This position statement is for all stakeholders who influence food and vegetable intake for children, aged 2–11 years, across a range of community settings, including, health, education, and sporting activities, including primary carers of children or parents, and the provision of food including food service and the food system supply chain.

## This focus on Australian children’s vegetable intake takes a three-component approach (with evidence to support these detailed below)

1. Shift emphasis from health to liking and enjoyment of vegetables
2. Create opportunities for vegetable exposure
3. Work smarter through collaborative action.

To remedy low vegetable intakes, a ‘child first’ approach is recommended, seeking to reach children in all early years’ settings where they may eat. Food preferences and habits are generally established in infancy and childhood and can persist throughout adulthood^(4–6)^. It is hard for adults to adopt new dietary habits, but children’s preferences are more malleable, and children can learn to like foods^(4,5)^.

## Position

In summary, our position is that children need to be repeatedly and consistently exposed to a variety of vegetables from an early age to achieve an increase in vegetable intake^(7)^. A focus on enjoyment and learning to like eating vegetables at an early age is critical to forming favourable lifelong eating habits^(4,5)^.

Coordinated work is needed to ensure vegetables are available and positively promoted in a range of settings, using evidence-based initiatives, thereby creating an environment that will support children’s acceptance of vegetables, in turn facilitating increased intake, and ultimately realising the associated health benefits^(8)^.

The challenges and evidence base for recommending a new approach are detailed below.

## Need for a paradigm shift to address the current challenges

Vegetables are the cornerstone of a healthy diet. Vegetable intake, as part of healthy eating, helps promote health and prevent disease, but despite the well-known health benefits, low vegetable intakes occur in Australian children^(1)^.

There have been significant efforts made to encourage children to eat more vegetables, but longer-term sustained success is modest at best^(1)^. There are several challenges that may have limited past efforts and interventions:

### Taste and other sensory properties

Children are driven by the appearance, taste, flavour, and texture, and not the healthiness of food^(9,10)^. The concept of health and the delay in reward to achieve healthiness are hard for children to understand. Children struggle to process multiple concepts simultaneously^(11)^. Health messages can evoke disliking and set an expectation of poor taste^(9)^.

Vegetables can taste bitter or bland^(10)^. These sensory characteristics of vegetables are not innately liked by children, and enjoyment of foods with these tastes may take time to develop^(9)^.

### Time and occasion of eating

Australian children eat most of their vegetables within their dinner meal, and including vegetables and vegetable products within other meals or as snacks throughout the day is not common^(9,12–14)^ This is problematic because the number of eating occasions is insufficient to facilitate familiarity, exposure, quantity and variety of consumption opportunities.

### Dietary advice

Recommendations for healthy eating tend to be broad and lack action-orientated advice. Fruit and vegetables have commonly been combined in recommendations and interventions^(2,15,16)^, but they taste different, and are eaten in different ways at different times. When combined with fruit, fruit intakes tend to increase but not vegetables^(2)^. Encouraging vegetable intake requires specific vegetable strategies and messaging.

### Distribution and access

There are supply and distribution challenges in getting a regular supply of vegetables and vegetable products onto the daily menus of early childhood education and care (ECEC) and schools^(17)^.

### Marketing and promotion

Vegetables and vegetable products are not marketed as widely as other products that are less healthy but more appealing to children^(18–20)^.

### Stakeholder coordination

Coordination of efforts is challenging because motivated stakeholders such as growers, retailers, ECEC providers, educators, health scientists and public health specialists are disconnected but ultimately have the same goal of greater intake of vegetables by Australian children^(21)^. There is little coordination or connection at national/state levels, and there is no mandate to connect. There is disparity between the sectors and their ideal outcomes.

## The new paradigm solution

Evidence suggests that greater success could be achieved through a coordinated shift in focus towards teaching and supporting children to become familiar with, accept and like vegetables through increased exposure to a greater variety of vegetables and vegetable products from an early age. There are three focus areas.

### Shifting focus to liking and enjoyment of vegetables

The first focus area calls for change in the message to enjoyment (and not health as an outcome) through vegetable-specific guidelines and the mechanism of repeated exposure to vegetables. Opportunities for action include:

1. Implement initiatives to emphasise and encourage children’s enjoyment of eating vegetables in multiple settings and through curriculum activities, for example, in day care, pre-school (ECEC) and school^(9,22,23)^.
2. Provide practical advice to parents^(24)^, educators and carers on repeated exposure to new vegetables and positive reinforcement of vegetable tasting by children^(7,23)^.
3. Provide evidence of best practice to decision-makers to help shape existing and future dietary recommendations, and guidelines or infrastructure about repeated exposure and variety as strategies to increase children’s liking of vegetables across life stages and in all settings where children eat food^(23)^.
4. De-emphasise explicit health messages^(9)^.
5. Consider including framing messages that refer to the low environmental impacts of plant-based diets to encourage a positive response from providers (seeking to lower their carbon footprint) and carers (‘green’ values)^(25)^.
6. Incorporate innovative techniques in food marketing and apply them to vegetables^(26)^.

### Create vegetable exposure opportunities for children

The second focus area calls for an increased availability at children’s eating occasions, increasing the ease of vegetable supply to children’s settings to facilitate children’s involvement and familiarity with vegetables. Opportunities for action include:

1. Incorporate evidence that early introduction and repeated exposure, particularly to a variety of vegetables, would encourage intakes^(9,23)^.
2. Develop new vegetable snacking occasions, so children can more easily consume vegetables for snacks, out of the home (i.e. in lunch boxes) or at any time.
3. Develop new vegetable products, so children can more easily consume vegetables for snacks, out of the home (i.e. in lunch boxes) or at any time^(27)^.
4. Support caterers in settings (schools, ECEC, and hospitals) through initiatives such as new tasty vegetable products, direct delivery, and chefs’ and carers’ training^(17)^. Provide support at the organisational level through policy (food supply/distribution included).
5. Incentivise and reward the inclusion of vegetables and vegetable products in menus at schools, hospitals and ECEC through recognition and award, for example, five-star food service (analogous to health star ratings of foods).
6. Optimise the taste, flavour and texture qualities of vegetables and vegetable products according to children’s preferences through product development and preparation. Provide guidance, training and support to industry and food service for the development of vegetable-based products with high sensory appeal^(9,27)^.
7. Involve children in preparation and cultivation of vegetables at home, in school ^(28,29)^ and in childcare centres^(30)^ so that they have experiential familiarity of vegetables.
8. Initiate ‘incursions’ from growers into childcare and schools to increase children’s familiarity with the origins of vegetables and leverage children’s love of nature^(31)^.

### Working together

The third focus area suggests coordinated efforts using centralised resources to support every child’s opportunity to learn to like vegetables. Opportunities for action include:

1. Develop interrelated efforts across relevant food systems (distribution, food marketing, packaging, food relief and retail) to ensure all Australian children have access to a variety of vegetables^(21)^.
2. Create a coordinated delivery system that helps supply vegetables from farms to children via ECEC and school settings^(17)^. For example, delivered produce boxes supported by training for educators, carers and cooks^(17,32)^.
3. Strengthen school canteens, ECEC and other food services policies^(33,34)^ and create national school canteen and food service policy guidelines to include serves of vegetables in settings where at least one meal or snack is provided to children aged 2–11 years, that is, school canteens, Out of School Hours Care, ECEC, sporting facilities, food service outlets, etc.
4. Create a sustainable model of coordination across settings and stakeholders, for example, through integration into Australian State Government Health and Wellbeing policy and initiatives, and Australian initiatives such as The Fruit and Vegetable Consortium and the National Nutrition Network^(26,34,35)^.
5. Australian children are supported to learn to like a variety of vegetables, with resources available for multiple settings (e.g. home, ECEC and school). Additional assistance may be required for remote regions that cannot receive fresh vegetables in having access to frozen or healthy processed forms of vegetables. Provision in ECEC and schools reduce the cost burden of food purchase for low-income families.
6. Create a centralised hub of information for stakeholders. The hub could contain resources such as vegetable-based recipes, video content on repeated exposure training for introducing vegetables for families, introduction of vegetables as a first food for new parents, snack and lunch box vegetable ideas, and education about vegetables from paddock to plate. The greater project this statement has emerged from is a beginning (www.vegkit.com.au).

## Discussion

This position statement has been a collective effort by experts and stakeholders in the domain and represents a novel health promotion approach to a perennial problem. Evidence suggests greater success could be achieved through a coordinated shift in focus towards teaching and supporting children to become familiar with, accept and like vegetables through increased exposure to a greater variety of vegetables and vegetable products from an early age, facilitated by coordinated action in all settings in which children eat.

Low vegetable intakes prevail in many OECD countries and strategies suggested here may have universal application in out-of-home settings. Additional sustained work is needed in home settings^(3,11,13,24)^ including an exposure/liking paradigm.

This consensus document was agreed and authored by academics and practitioners in public health, and retail. Furthermore, nine other stakeholder sectors, including health, education, ECEC and industry supply chain, agreed to be acknowledged for supporting the statement.

This statement pertains to the Australian environment where meal provision in state schools is uncommon, in contrast to many other jurisdictions. However, the same principles expressed in the statement would apply to other supply channels such as school meals. In Australia, challenges to canteen supply and food brought from home are similar, with an absence of vegetables in both^(36)^. Trials undertaken, as part of the larger project in which the current work was located, succeeded in increasing vegetable sales in school canteens^(37)^. Opportunities in ECEC across OECD countries may be universal.

There are extra challenges in rural and remote locations that require additional effort in supply and appropriate inter-personal factors, such as community involvement and role modelling, to provide opportunity for rural and remote children^(38)^.

Further work requires a call to action to stakeholders with both interest and influence in specific settings and jurisdictions. For example, this includes consideration in current revisions of the Australian Dietary Guidelines (the cited review by Bell et al.^(7)^, awaits submission to this process), and possible implementation of the recently released Australian National Obesity Strategy^(39)^, National Preventive Health Strategy^(40)^ and non-government initiatives, such as the Fruit and Vegetable Consortium^(26)^ that is seeking to fill a gap.

## Conclusion

In summary, children need to be repeatedly and consistently exposed to a variety of vegetables from an early age to achieve an increase in vegetable intake. Learning to like eating vegetables at an early age is critical to healthy diets. Coordinated work on availability and promotion across settings, using evidence-based initiatives, will create an environment that will support children’s acceptance of vegetables, increased intake and associated health benefits.
